# Supplementary material for: Limits on information transduction through amplitude and frequency regulation of transcription factor activity
Source: eLife. 2015 May 18;4:e06559. doi: 10.7554/eLife.06559 (PMC4468373; doi:10.7554/eLife.06559)
Supplement: Supplementary file 1. — Raw single-cell time-trace data for HXK1 (15259 cells), SIP18 (21242 cells), pSIP18 mut A (18203 cells), pSIP18 mut B (17655 cells), 1× reporter diploid (21236 cells), and 2× reporter diploid (19222 cells). The data are also available from Dryad Digital Repository (Hansen and O'Shea, 2015). DOI: http://dx.doi.org/10.7554/eLife.06559.014 [file elife06559s001.zip › source_data/source_data_READ_ME.pdf]

READ ME file for source data accompanying

Limits on information transduction through regulation of signaling dynamics

Anders S. Hansen & Erin K. O'Shea

This file describes the organization of the source data accompanying the manuscript "Limits on information transduction through regulation of signaling dynamics".

All source data is given as comma separated value files (csv). There are 103 data files in csv format, one for each experiment for each promoter. For example, "SIP18_FM7_786minINT_690nM.txt" contains raw data for the *SIP18* promoter where the Msn2 input consists of seven 5-min pulses of Msn2 separated by 7.86 min intervals. Likewise, "2x_SIP18_HXK1_FM4_175minINT_690nM.txt" contains data for the 2x reporter diploid (2x *sip18*::YFP & 2x *hxk1*::CFP), where the Msn2 input is four 5-min pulses separated by 17.5 min intervals. As a further example, "pSIP18_mutA_DM_70min_1117nM.txt" contains data for *pSIP18* mut A, where the Msn2 input is a single pulse (hence "DM") of 70 min duration. Finally, "SIP18_info_theory_untreated.txt" contains *SIP18* data for the untreated experiment – that is, without 1-NM-PP1 treatment.

Each csv file file contains *n* rows and 128 columns, where *n* is the number of cells for that particular experiment. In general, we have data for around ~1000 single cells for each experiment so most csv file have around ~1000 rows. Each row contains YFP (mCitrineV163A) and CFP (SCFP3A) data for a single cell. Columns 1 to 64, inclusive, contain the YFP time-series data and columns 65-128, inclusive, contain the CFP time-series data for that particular cell. The 64 columns correspond to the 64 frames collected in the time-lapse microscopy experiment and correspond to a time vector running from [-5, -2.5, 0, 2.5, …, 152.5] in units of minutes and 2.5 min intervals. Msn2 is always activated for the first time at time-point '0 min'. YFP and CFP data is in arbitrary fluorescence units and is the average fluorescence intensity in a particular cell (i.e. the mean pixel intensity averaged over the entire cell).

The 1-NM-PP1 concentrations used for the amplitude modulation experiments (100 nM, 175 nM, 275 nM, 413 nM, 690 nM, 1117 nM and 3 μM) correspond roughly to 25%, 37.5%, 50%, 62.5%, 75%, 87.5% and 100% of maximal nuclear Msn2 localization. The concentration 690 nM was used for all oscillatory experiments.

The high-resolution Msn2-mCherry input traces used in Figure 2 to show the Msn2 input where obtained by fitting to the raw, measured Msn2-mCherry and they are given in the file entitled "fitted_msn2_traces.txt". The first row in file "fitted_msn2_traces.txt" contains the time vector running from -5 min to 152.5 min, in short intervals of 0.05 min. The subsequent rows contain DM Msn2 input running from 100 nM to 3 μM and the amplitude is given as a fraction of the maximal input. After the DM rows, the final rows contain the FM data starting from FM1_5min running until FM9_5minINT.

The source data given here is raw, but corrected for photobleaching. For the dual reporter strains, *orf*::YFP/*orf*::CFP (*HXK1*, *SIP18*, *pSIP18* mut A and *pSIP18* mut B), the CFP and YFP values may need to be further rescaled because although biological mean (i.e. number of proteins per cell) are the same, the measured YFP and CFP values are not since the fluorescent proteins have different brightness levels, different exposure times were used, the microscope LEDs for YFP and CFP are different and a host of

other technical reasons. We note the information theoretical calculations of mutual information are unaffected by rescaling.

We also note that we have slightly changed the image analysis and microscope excitation setup, so that the AU values are slightly different, in an absolute sense, compared to our previous data set (Hansen & O'Shea, 2013).

For the 1x and 2x reporter strains YFP reports on *SIP18* (*sip18*::YFP) and CFP reports on *HXK1* (*hxk1*::CFP) and the data should therefore not be rescaled.

Finally, we note that YFP is a better fluorescent protein than CFP and exhibits higher signal-to-noise – it is substantially brighter and under our experimental conditions the cells exhibit much lower autofluorescence background in the YFP part of the spectrum. Correcting for and subtracting out autofluorescence is especially challenging at low expression levels where the actual gene expression signal might be significantly lower than the autofluorescence background, such that any fluctuations in autofluorescence becomes hard to distinguish from changes in the actual gene expressions signal. This is much less of a problem for YFP where the signal-to-noise is good enough for extremely precise quantification of even very low gene expression (~ 100 AU), but for CFP, quantification of low gene expression (e.g. below ~500 AU) is challenging and the data more noisy. This is why we use the YFP data to perform calculations of mutual information.
