# Supplementary material for: Limits on information transduction through amplitude and frequency regulation of transcription factor activity
Source: eLife. 2015 May 18;4:e06559. doi: 10.7554/eLife.06559 (PMC4468373; doi:10.7554/eLife.06559)
Supplement: Supplementary file 2. — Computation of Mutual Information. Complete description of information theoretical computations and the algorithm. DOI: http://dx.doi.org/10.7554/eLife.06559.015 [file elife06559s002.pdf]

SUPPLEMENTARY FILE 2
COMPUTATION OF MUTUAL INFORMATION

ANDERS S. HANSEN & ERIN K. O'SHEA

Contents

1. Introduction 2
2. Mutual information for a single reporter 2
2.1. Shannon entropy and mutual information for a single reporter 2
2.2. Maximal mutual information for a single reporter 2
2.3. Computing mutual information with empirical data 3
2.4. Comments on how to interpret maximal mutual information 4
2.5. Comments on using a scalar for computing mutual information 5
2.6. Comments on applying the Data Processing Inequality 6
3. Joint mutual information for two reporters 9
3.1. Joint mutual information for two reporters 9
3.2. Joint maximal mutual information for two reporters 9
3.3. Computing joint mutual information with empirical data 10
4. Determining intrinsic mutual information 11
4.1. Intrinsic and extrinsic contributions to gene expression noise 11
4.2. Effect of extrinsic noise on mutual information 12
4.3. An algorithm to estimate the intrinsic mutual information 12
4.4. Testing the algorithm with simulated data and linear gene expression models 14
4.5. Testing the algorithm with simulated data and nonlinear gene expression models 17
4.6. Comments on the algorithm 17
4.7. Computing I_{int} with empirical data 18
5. References 18

1. Introduction

In this document, we describe in detail how all information theoretical calculations are performed. In Section 2, we define mutual information and explain how it is computed with empirical data and adjusted for bias. We further discuss how to interpret mutual information. In Section 3, we describe how joint mutual information, in the case where we consider two reporters, is computed. In Section 4, we describe the algorithm that we use to estimate the intrinsic mutual information. We also go through the steps used to test and verify the algorithm *in silico*. Finally, Section 5 contains references.

2. Mutual information for a single reporter

2.1. **Shannon entropy and mutual information for a single reporter.** We consider the transcription factor (TF) input - gene expression output relationship for a dose-response type relationship. E.g. we consider the YFP gene expression response to different amplitudes or frequencies of TF input in single cells. For each cell we quantify the YFP gene expression response as a scalar and then bin the responses into appropriately sized bins such that we have a discrete probability distribution (Response output) for each TF input (Signal).

Considering all signals (all TF inputs), we can calculate the total Shannon entropy (H) of the responses (R) by summing over all bins i:

$$H(R) = -\sum_i P(R_i)\log_2(R_i) \tag{2.1}$$

Next, to proceed towards defining mutual information, we compute the conditional entropy of the YFP response given the signal

$$H(R|S) = -\sum_j P(S=s_j)\left(\sum_i P(R=r_i|S=s_j)\log_2(P(R=r_i|S=s_j))\right) \tag{2.2}$$

Then finally, mutual information in bits is given by:

$$MI(R;S) = H(R) - H(R|S) \tag{2.3}$$

2.2. **Maximal mutual information for a single reporter.** The mutual information is a function of the signal distribution, $P(S)$. To determine the maximal mutual information, $I(R;S)$, we need to find the signal distribution, $P(S)$, that maximizes the mutual information, $MI(R;S)$. In other words, the maximal mutual information, I, is defined as:

$$I(R;S) = \max_{P(S)}[MI(R;S)] \quad \text{for} \quad \sum_i P(S_i) = 1; \quad P(S_i) \geq 0 \tag{2.4}$$

This is an optimization problem and $P(S)$ can be numerically determined such that the maximal mutual information, $I(R;S) = \max_{P(S)}[MI(R;S)]$, is obtained using the iterative Blahut-Arimoto algorithm (Blahut, 1972; Arimoto, 1972). The maximal mutual information can be thought of as the information capacity and is the upper bound on mutual information.

2.3. **Computing mutual information with empirical data.** To calculate the maximal mutual information for individual promoters with respect to amplitude or frequency modulation, we must first discretize the data. For each promoter we obtain single-cell time-lapse responses to each signal input (e.g. amplitude of Msn2 or frequency of Msn2). To convert the single-cell time-series into a scalar, we smooth the YFP time-series using moving-average smoothing to minimize the effect of technical noise. All single-reporter analysis uses YFP reporter expression rather than CFP reporter expression, since YFP suffers from lower technical noise. Following smoothing, the maximal YFP value for each single cell is used for calculating mutual information. Thus, the time-series is converted into a scalar.

Calculations of maximal mutual information, I, using discretized data are biased by binning and undersampling (Cheong *et al,* 2011). However, for each promoter we are able to obtain 15,000-20,000 single cell responses yielding on average more than 1,000 single cell responses per condition. Thus, for the single-reporter calculations we are far away from the seriously undersampled regime. Likewise, binning can introduce bias (Figure 1). If the data is binned too coarsely, differences in the single cell responses are no longer adequately captured and the estimated I is an underestimate. Conversely, if the data is binned into so many bins that there are not enough cells to accurately estimate the probability density of each response bin, undersampling becomes important and the estimated I is an overestimate.

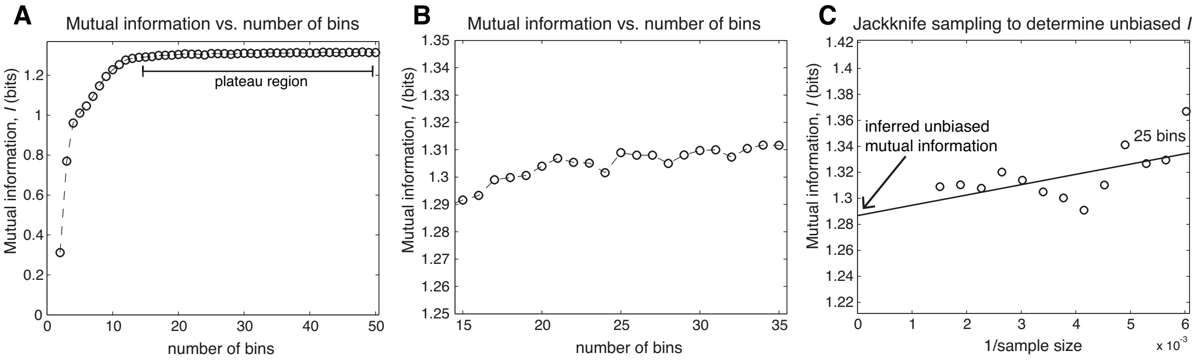

FIGURE 1. **Correcting I for bias**
A) Using *HXK1* I_{AM} as an example, how I scales with the number of bins is plotted. After around 12-14 bins, a plateau region is reached. **B)** A zoom-in plot of **A)** from 15 to 35 bins. **C)** An illustration of how jackknife sampling is used to correct for undersampling and infer an unbiased I.

Methods are available to deal with both the issue of binning and undersampling. Here we largely follow the approach used previously by Cheong, Levchenko and co-workers (Cheong *et al,* 2011) and also described by Bialek and co-workers (Strong *et al,* 1998; Slonim *et al,* 2005). This method is also known as the "direct method". To determine the optimal number of bins, we plot I as a function of the number of bins (Figure 1). As shown in Figure 1A, below 10 bins the binning is so coarse that substantial information is lost and I is a serious underestimate. After around 12-14 bins, I reaches a plateau. I should be calculated in the plateau region (Cheong *et al,* 2011; Slonim *et al,* 2005). For all single reporter calculations of I, we follow the approach of Cheong *et al.* and report I_{AM} and I_{FM} for a promoter as the mean I calculated using 15 to 35 bins (the region shown in Figure 1B), inclusive, and the error in the I estimate as the standard deviation using 15 to 35 bins, inclusive. The only exception is *SIP18* where we used 15 to 25 bins, inclusive.

To correct for undersampling, we consider a series expansion of I in terms of inverse powers of sample size, N, (Cheong *et al,* 2011):

$$I_{\text{biased}} = I_{\text{unbiased}} + \frac{k_1}{N} + \frac{k_2}{N^2} + ... \approx I_{\text{unbiased}} + \frac{k_1}{N} \tag{2.5}$$

where I_{unbiased} is the desired maximal mutual information corrected for undersampling, N is the number of cells per condition (sample size) and k_i are coefficients. Since we have on average around or above 1000 cells per conditions, we can ignore all terms of second order and higher and I_{unbiased} is therefore approximately a linear function of the inverse sample size. We estimate the linear function in equation (2.5) using jackknife sampling as has been done previously (Cheong *et al,* 2011). We sampled fractions of the data ranging from 100% to 25% without replacement and computed I in each case by optimizing $P(S)$ using the Blahut-Arimoto algorithm to obtain the maximal mutual information. Figure 1C shows an example using *HXK1* data and 25 bins. As is evident, even with 25 bins, we have so many single cell responses that undersampling is minimal and the difference between I_{unbiased} and I_{biased} is only ~ 0.02 bits.

As mentioned above, to determine I_{AM} and I_{FM} of individual promoters we perform jackknife sampling to estimate C_{unbiased} using 15 to 35 bins, inclusive, and report I as the mean and the error as the standard deviation. In the case of *SIP18* and *HXK1*, we can also estimate the error in our measurement by comparing I_{AM} and I_{FM} obtained in the *sip18*::YFP/*sip18*::CFP and *hxk1*::YFP/*hxk1*::CFP strains with I_{AM} and I_{FM} obtained from the 1x *sip18*::YFP/*hxk1*::CFP strain:

I	*gene*::YFP/*gene*::CFP strain	1x *sip18*::YFP/*hxk1*::CFP strain
I_{AM}(*sip18*::YFP; S)	1.21 ± 0.03 bits	1.17 ± 0.02 bits
I_{FM}(*sip18*::YFP; S)	0.52 ± 0.06 bits	0.50 ± 0.05 bits
I_{AM}(*hxk1*::CFP/YFP; S)	1.30 ± 0.01 bits	1.30 ± 0.01 bits
I_{FM}(*hxk1*::CFP/YFP; S)	1.11 ± 0.01 bits	1.14 ± 0.01 bits

Here AM refers to the maximal mutual information with respect to amplitude modulation and FM refers to the maximal mutual information with respect to frequency modulation. As is evident from the table above, the estimates of I_{AM} and I_{FM} between strains are very similar. We note that these are different yeast strains in slightly different genetic backgrounds. Thus, the fact that the *SIP18* and *HXK1* I_{AM} and I_{FM} values have such low estimated errors and agree so well between different yeast strains in different genetic backgrounds measured during independent experiments, gives us high confidence in the measurements. Furthermore, this shows that measurements of I for natural genes are robust between different clones and robust to slight variation in genetic background.

2.4. **Comments on how to interpret maximal mutual information.** Information theory was developed by Claude Shannon as a mathematical theory of communication to quantify information transmission for engineering applications (Shannon, 1948; Cover & Thomas, 2006). Accordingly, the concept of a channel capacity was originally introduced for a discrete, memoryless channel and is precisely defined in information theory. Here we consider the TF input - YFP output relationship as a noisy channel in the sense that each individual input is converted to a noisy distribution of YFP output. This TF-YFP channel differs from the discrete memoryless channel considered by Shannon in several important ways (Bowsher & Swain, 2014). For example, the TF-YFP channel is a single-use channel, whereas a communication channel is used repeatedly. In fact, one of the central results in information theory, the noisy-channel coding theorem, applies only to repeated use and states that all rates, R, (e.g. in bits per second) below channel capacity, C, are achievable. Thus, the noisy channel coding theorem and other theorems in information theory do not hold in

our case. Furthermore, as is clear from equation (2.3), the mutual information depends on $P(S)$, the distribution of signals or inputs. While in engineering applications the distribution of inputs that maximizes the mutual information can conceivably be chosen by the user, in the case of a cell responding to environmental perturbations like stress or changes in hormone or growth factor levels, $P(S)$ is presumably experienced rather than chosen by the cell. Thus, while the channel capacity, C, is defined as the maximal mutual information, we refer to the quantity as maximal mutual information or I in this study. I still provides an upper bound on the maximal mutual information transmissible and should be interpreted as such. We choose not to refer to it as mutual information, because mutual information has a $P(S)$-dependence. Similarly, we choose not to refer to it as channel capacity because channel capacity has a precise definition in the context of engineering applications that does not, strictly speaking, apply in cellular signaling. Furthermore, Shannon considered the transmission of discrete signals, whereas here we consider dynamic signal transduction. In other words, we consider information transmission between a dynamic input signal ([Msn2(t)]) and a dynamic output response ([YFP(t)]). When applying concepts from information theory such as the Data Processing Inequality (DPI) to dynamic signaling events, care has to be taken. As we will discuss in further detail in the next sections, if scalars are used to represent dynamic signals this can lead to an underestimation of the actual amount of information transduced.

Given these caveats, how then should we interpret the calculations of I? Entropy can be thought of as a measure of uncertainty and mutual information quantifies the reduction in uncertainty about the input (e.g. the TF amplitude) that is gained from measuring the output (e.g. YFP level). Thus, the calculated I is the maximal reduction in uncertainty about the TF input that can be gained by measuring the YFP output. This is often interpreted in terms of distinguishable states such that e.g. $I = \log_2(3) = 1.59$ bits means that the cell can distinguish, without error, three different inputs (Bowsher & Swain, 2014; Levchenko & Nemenman, 2014). However, this is not always the case. For example, $I = 1.59$ bits, does not guarantee that the cell can distinguish three different inputs without error. Similarly, it is possible to have a situation where it is possible to distinguish three different inputs with a low error, even though I is much less than 1.59 bits.

To illustrate this, consider the distributions in Figure 2 (this example is adapted from Figure 1 in (Bowsher & Swain, 2014)). As shown in this example (Figure 2), even $I = 0.61$ bits allows some inference about the input from observing the output. In Figure 2B, $I \geq 1$ bits, but no two inputs can be distinguished without error. The overlap between the blue and red distributions is small and thus the inference error will be small, but this does illustrate that $I \geq 1$ bits, does not necessarily allow for distinguishing two inputs without error. Likewise, even though $I \sim 1$ bits and not 1.59 bits ($\log_2(3)$) in Figure 2B, it is clearly possible to distinguish the three inputs, albeit with significant error. Therefore, when interpreting the value of I it is important to also consider the shape of the probability distributions. Finally, comparing Figure 2C and Figure 2D, we see that even though the difference in I is only 0.02 bits, the difference in the output variability given the input is quite high. More lengthy discussions on how to interpret bits are given in (Bowsher & Swain, 2014; Levchenko & Nemenman, 2014).

2.5. **Comments on using a scalar for computing mutual information.** For each single cell we measure a YFP time-trace. Specifically, the YFP level is measured 64 times in each single cell during an experiment at 2.5 min intervals. For all information theoretical calculations we use a YFP scalar rather than a YFP vector. We use a moving-average filter to smooth the YFP trace and then pick the maximal YFP value after the YFP trace has reached a plateau. Smoothing and picking a single value greatly reduces effects from measurement noise. However, the shape of the YFP trace also contains information: e.g. when the YFP trace begins to rise, how steeply it increases, when it reaches a plateau etc. If all of these effects were included in the calculations, the calculated I

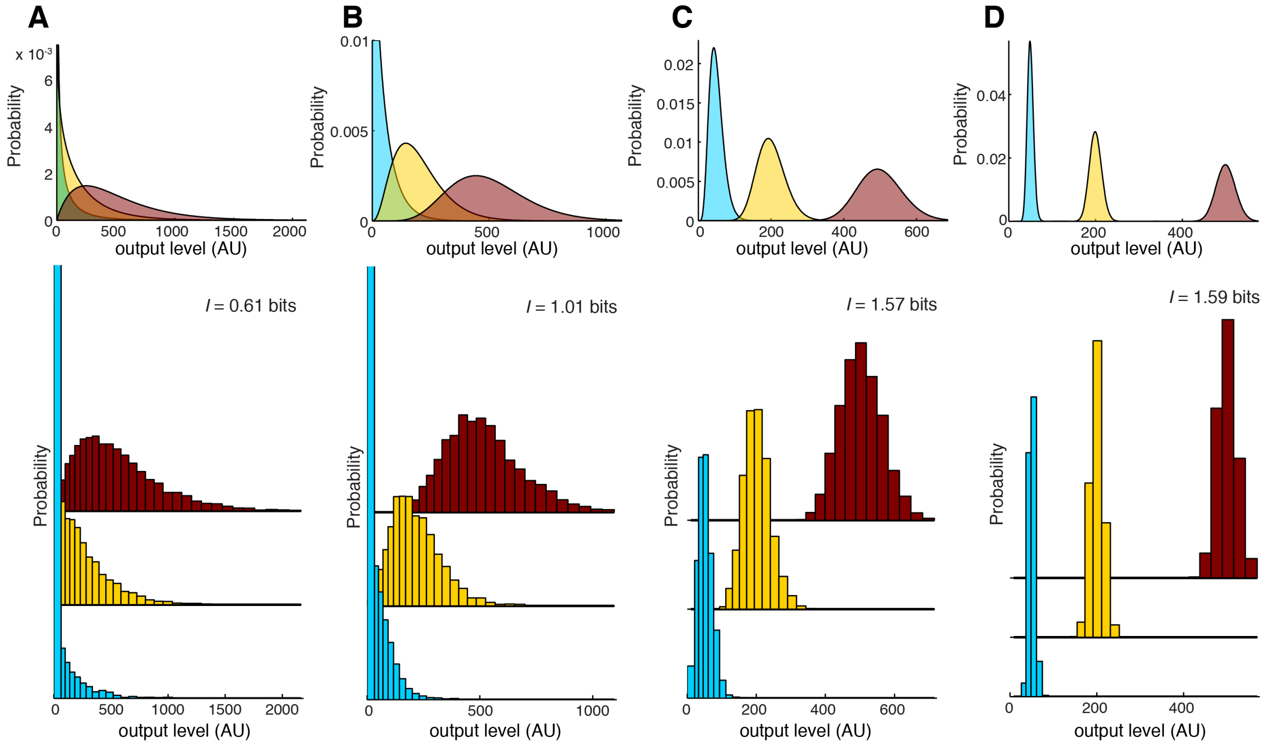

FIGURE 2. **Interpreting I**
Illustration of how to interpret I. Gamma distributions were simulated with different variances. The means are 50 (blue), 200 (yellow) and 500 (red) in all cases, but the variances were different. In each case, I was calculated. The gamma distribution b values were 250 **(A)**, 56 **(B)**, 7.5 **(C)** and 1 **(D)**.

would be higher. However, we posit that the max YFP level, that is, the YFP level after the time-trace has reached a plateau, is the most biologically relevant quantity. When a cell is exposed to a particular stress or signal, the goal is presumably to make the right protein in the right amount. In all likelihood, the shape of the protein production rate curve matters much less than the final level of the protein. Thus, for all information theoretical calculations in this work we use only the final maximal YFP level.

Nevertheless, we did consider how alternative quantification schemes would affect calculations of I. The simplest alternative measure is to use the YFP level at a specific timepoint. However, as shown in Figure 3 if we use YFP at 120 min (Figure 3B) or at 150 min (Figure 3C), this has a minimal effect on I_{AM} compared to using the maximal YFP value (Figure 3A). Another alternative, is to the use the YFP production rate, i.e. $\frac{\mathrm{d\ YFP}}{\mathrm{d}\ t}$. For this calculation we use the maximal YFP production rate (i.e. the rate when $\frac{\mathrm{d^2\ YFP}}{\mathrm{d}\ t^2} = 0$). As shown in Figure 3D, if the production rate is used for calculation of I, we get a much lower value of I. This is consistent with the production rate being a poorer measure of gene expression.

2.6. **Comments on applying the Data Processing Inequality.** A central result in information theory and a major motivation behind this work is the Data Processing Inequality (DPI). The DPI states that in a network of transmission events, once information has been lost, it cannot be recovered

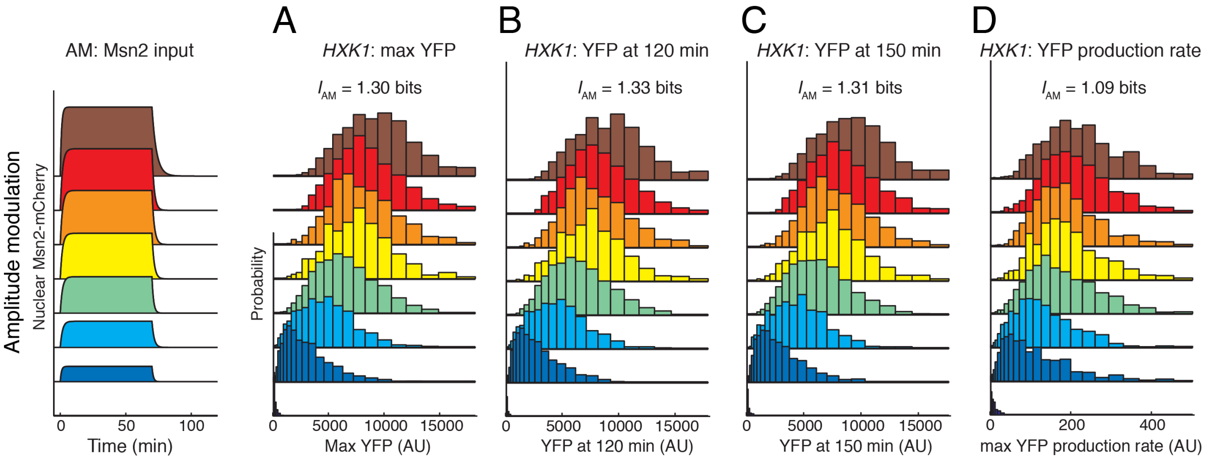

FIGURE 3. **Alternative YFP measures for calculating** I
To illustrate different methods for calculating I we use *HXK1* with respect to amplitude modulation as an example. **A)** Calculating I_{AM} using max YFP. **B)** Calculating I_{AM} using the YFP value at 120 min. **C)** Calculating I_{AM} using the YFP value at 150 min. **D)** Calculating I_{AM} using the maximal YFP production rate. We define the maximal production rate as the average rate of YFP production from 5 min before to 5 min after the maximal rate. The maximal rate timepoint was determined by differentiating the YFP trace after smoothing.

as is also observed in a game of 'broken telephone'. In other words, post-processing cannot increase information.

More formally, consider a Markov chain $X_1 \to X_2 \to X_3$. The DPI states that if X_2 is already known, then observing X_3 provides no additional information about X_1 and observing X_1 gives no additional information about X_3. That is:

$$I(X_1; X_3) \leq I(X_1; X_2) \tag{2.6}$$

More generally, for a Markov chain $X_1 \to X_2 \to X_3 \to X_4 \to ... \to X_n$, the DPI states that:

$$I(X_1; X_n) \leq ... \leq I(X_1; X_4) \leq I(X_1; X_3) \leq I(X_1; X_2) \tag{2.7}$$

In the context of our work, we use the DPI to argue that it does not matter how much information can be encoded and transduced upstream during stress exposure. Assuming that the purpose of the cell is to make the right stress response protein in the right amount, measuring YFP levels provides an overall measure of the information transduction of the entire pathway. In other words, even if high information transduction upstream of Msn2 is observed ($I(\text{Msn2}; X)$), the overall pathway cannot transduce more information than the final decoding step ($I(\text{YFP}; \text{Msn2})$. Thus, according to the DPI a pathway cannot transmit more information than the capacity of its 'weakest link'. Therefore, by quantifying information transduction across a pathway's weakest link, we can place an upper limit on information transduction across the entire pathway.

However, while the DPI is very intuitive, its application to dynamic networks is more complicated. To understand why, consider the central dogma TF $\to$ mRNA $\to$ YFP, where we use

YFP to denote the protein being produced and a TF is activating the gene. According to the DPI, $I(\text{TF};\text{YFP}) \leq I(\text{TF};\text{mRNA})$. However, if snapshot measurements of TF, mRNA and YFP are used, this can cause an apparent breakdown of the DPI if the mRNA has a much shorter life-time than the protein (which is typically the case in a cell). For example, if the mRNA has a lifetime of a few minutes and the protein a lifetime of several hours, a snapshot measurement of the mRNA reflects the transcription activity during recent minutes, whereas the protein reflects the integrated mRNA level over the past few hours. To illustrate this point, consider Figure 4, which shows two stochastic simulation runs for the standard random telegraph gene expression model. As can be seen, snapshot measurements of mRNA copy number are much less reliable measurements of total promoter activity than is the protein copy number after its plateau (after ∼75 min).

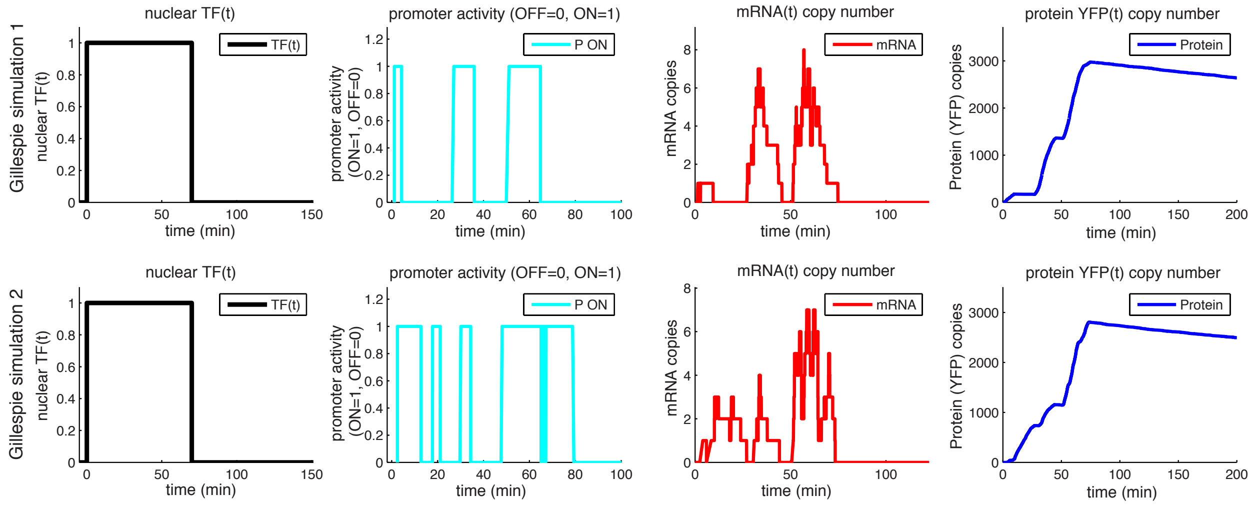

FIGURE 4. Stochastic simulation of the random telegraph model
To illustrate the importance of dynamics, consider two simulation runs of the standard random telegraph gene expression model. First panel, a 70 min pulse of nuclear TF. Second panel, when the promoter is active (ON) as a function of time. Third panel, mRNA copy number as a function of time. Fourth panel, protein copy number as a function of time. Two simulations runs are shown for comparison.

Therefore, if snapshot measurements were used to characterize the mRNA and YFP distributions under different levels of TF, it would be possible to observe $I(\text{TF};\text{YFP}) \geq I(\text{TF};\text{mRNA})$ in apparent violation of the DPI. Of course, the DPI always applies, but for a dynamic pathway the time-dimension must be included: $\text{TF}(t) \rightarrow \text{mRNA}(t) \rightarrow \text{YFP}(t)$. In other words, had the full time trajectory been included in calculations of mutual information, $I(\text{TF};\text{YFP}) \leq I(\text{TF};\text{mRNA})$ would hold as expected.

This highlights an important distinction between how much information a response contains and which part of the response actually provides information that is useful to the cell. As discussed above, we use a scalar to quantify the maximal YFP concentration after it has reached its plateau. If we had also included the shape of the YFP time-trace, higher information would have been measured. But as mentioned, we posit that the maximal YFP concentration is the biologically relevant quantity. In particular, for a stress response TF such as Msn2 which generally induces proteins that are enzymes rather than regulators, the downstream effects (e.g. enzymatic activity) is determined by protein concentration alone. Therefore, applying the DPI we can establish an upper bound on the pathway's ability to regulate the YFP concentration of a single gene.

This holds provided that the YFP concentration is the correct and biologically relevant measure. If a downstream decoding module existed that could read out the dynamics of YFP production, just using the YFP concentration would underestimate information transduction. Therefore, our claim that we have established an upper limit on the promoter information transduction capacity of a single gene holds if, and only if, our assumption that the biologically relevant output measure is the maximal protein concentration is correct.

3. Joint mutual information for two reporters

3.1. **Joint mutual information for two reporters.** Next, we consider the case where we have two different reporters for two different genes in the same cell responding to the same TF input signal. E.g. now we have *gene1*::YFP (R_1) and *gene2*::CFP (R_2). We can calculate the mutual information and I for each individual reporter as described above, but here we are interested in how much additional information can be transmitted when we consider not just one, but both reporters in the same cell. E.g. if we already know *gene1*::YFP, how much additional information do we obtain from the additional channel, *gene2*::CFP.

Applying the chain rule for mutual information, the mutual information of two reporters responding to the same signal is given by:

$$MI\,(R_1, R_2; S) = MI\,(R_1; S) + MI\,(R_2; S|R_1) \tag{3.1}$$

Or equivalently:

$$MI\,(R_1, R_2; S) = MI\,(R_2; S) + MI\,(R_1; S|R_2) \tag{3.2}$$

Considering equation (3.1) we already know the single reporter mutual information $MI\,(R_1; S)$ from equation (2.3). Thus, only $MI\,(R_2; S|R_1)$ remains to be determined. $MI\,(R_2; S|R_1)$ represents the additional information we obtain, already knowing $MI\,(R_1; S)$, from also knowing the response of the second reporter, R_2.

To compute $MI\,(R_2; S|R_1)$, we use:

$$\begin{aligned} MI\,(R_2; S|R_1) = &\sum_i \sum_j \sum_k P\,(R_1 = r_i, R_2 = r_j, S = s_k) \\ &\cdot \log_2 \left(\frac{P\,(R_1 = r_i)\,P\,(R_1 = r_i, R_2 = r_j, S = s_k)}{P\,(R_1 = r_i, R_2 = r_j)\,P\,(R_1 = r_i, S = s_k)} \right) \end{aligned} \tag{3.3}$$

Thus, from equations (2.3) and (3.3), we can calculate the additional mutual information gain from an extra reporter according to (3.1).

3.2. **Joint maximal mutual information for two reporters.** As for a single reporter (equation (2.4)), the maximal joint mutual information is defined as:

$$I\,(R_1, R_2; S) = \max_{P(S)} \left[MI\,(R_1, R_2; S) \right] \quad \text{for} \quad \sum_i P(S_i) = 1; \quad P(S_i) \geq 0 \tag{3.4}$$

Whereas before $P(R, S)$ was a 2D matrix, $P(R_1, R_2, S)$ is now a 3D matrix. To determine the maximal mutual information, $I\,(R_1, R_2; S)$, we consider the conditional response data, $P(R_1, R_2|S)$. As before, we need to find $P(S)$ that maximizes $I\,(R_1, R_2; S)$. To do so we convert $P(R_1, R_2|S)$ into a 2D matrix and, as before, apply the iterative Blahut-Arimoto algorithm (Blahut, 1972; Arimoto, 1972) to obtain $P(S)$ and hence $I\,(R_1, R_2; S)$.

3.3. **Computing joint mutual information with empirical data.** As in the single reporter case, we must correct for bias due to binning and undersampling. When considering two reporters, the issue of undersampling is rather more pertinent. If the number of bins for *sip18*::YFP (R_1) is n and the number of bins for *hxk1*::CFP (R_2) is also n, then the total number of bins is n^2 and hence, with the same number of total cells, the number of cells available for estimating the probability density of each bin is much smaller than in the single reporter case. Thus, calculations of mutual information are now prone to bias due to undersampling. To correct for bias, we follow the same approach as in the single reporter case (an example using the 1x reporter diploid with *sip18*::YFP *hxk1*::CFP is shown in Figure 5).

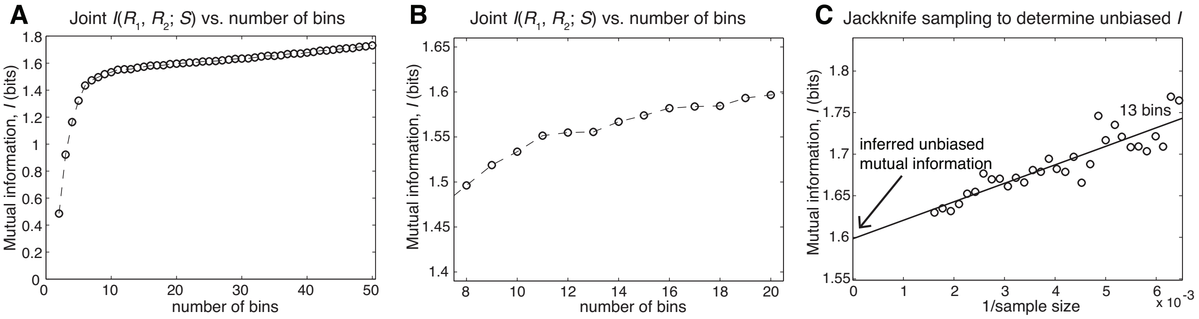

FIGURE 5. **Bias correction for joint mutual information, $I\left(R_1, R_2; S\right)$**
A) Using the 1x reporter diploid *sip18*::YFP *hxk1*::CFP joint maximal mutual information with respect to amplitude modulation as an example, how $I_{\text{AM, joint}}$ scales with the number of bins is plotted. **B)** A zoom-in plot of **A)** from 8 to 20 bins. **C)** An illustration of how jackknife sampling is used to correct for undersampling and infer an unbiased I_{joint}.

As shown in Figure 5A, for the joint $I\left(R_1, R_2; S\right)$, there is less of a plateau region compared to the single reporter $I\left(R; S\right)$ (Figure 1A). This indicates undersampling and is caused by the n^2 number of bins in the joint case. Nonetheless, we consider I_{joint} between 8 and 20 bins as shown in Figure 5B and calculate the joint $I\left(R_1, R_2; S\right)$ for 8 to 20 bins, inclusive, corrected for bias using jackknife sampling and the series expansion (Figure 5C and equation (2.5)) and report $I\left(R_1, R_2; S\right)$ as the mean and its error as the standard deviation. Thus, the approach is identical to the bias correction for a single reporter and essentially identical to previously reported bias correction approaches (Cheong *et al,* 2011). The results, after bias correction, are summarized in the table below:

I	1x *sip18*::YFP/*hxk1*::CFP strain	2x *sip18*::YFP/*hxk1*::CFP strain
$I_{\text{AM, joint}}\left(R_1, R_2; S\right)$	1.61 ± 0.04 bits	1.77 ± 0.04 bits
$I_{\text{FM, joint}}\left(R_1, R_2; S\right)$	1.18 ± 0.04 bits	1.26 ± 0.04 bits
$I_{\text{AM+FM, joint}}\left(R_1, R_2; S\right)$	1.67 ± 0.04 bits	1.83 ± 0.03 bits

Thus, having two copies of each gene instead of just one leads to a small, but robust increase in I_{joint}. Whereas $I_{\text{AM, joint}}\left(R_1, R_2; S\right)$ and $I_{\text{FM, joint}}\left(R_1, R_2; S\right)$ are for amplitude and frequency modulated input, respectively, $I_{\text{AM+FM, joint}}\left(R_1, R_2; S\right)$ is I when we combine all inputs and determine a single $P(S)$ that maximizes I over all inputs. Thus, $I_{\text{AM+FM, joint}}\left(R_1, R_2; S\right)$ is the information transduction capacity for amplitude and frequency modulated input combined.

Finally, we note that adding more promoter copies reduces the information loss due to intrinsic noise. We estimate this effect by comparing the 1x and 2x diploid results. But we can also make this comparison by calculating the joint mutual information between CFP and YFP in the dual reporter strains for *SIP18*, *HXK1*, *pSIP18* mut A and *pSIP18* mut B. For the calculations of

joint mutual information, we follow the same approach as described above. This also allows us to estimate the gain in I for *pSIP18* mut A and *pSIP18* mut B upon adding an additional promoter copy. The results are shown in the table below.

	I(YFP)	I(YFP,CFP)	I(1x CFP/YFP)	I(2x CFP/YFP)
SIP18 AM	1.21 ± 0.03 bits	1.29 ± 0.09 bits	1.17 ± 0.02 bits	1.26 ± 0.05 bits
SIP18 FM	0.52 ± 0.06 bits	0.59 ± 0.09 bits	0.50 ± 0.05 bits	0.53 ± 0.04 bits
HXK1 AM	1.30 ± 0.01 bits	1.40 ± 0.03 bits	1.30 ± 0.01 bits	1.47 ± 0.01 bits
HXK1 FM	1.11 ± 0.01 bits	1.20 ± 0.03 bits	1.14 ± 0.01 bits	1.20 ± 0.01 bits
mut A AM	1.42 ± 0.01 bits	1.53 ± 0.02 bits	n/a	n/a
mut A FM	0.88 ± 0.02 bits	1.02 ± 0.04 bits	n/a	n/a
mut B AM	1.55 ± 0.01 bits	1.61 ± 0.02 bits	n/a	n/a
mut B FM	1.39 ± 0.01 bits	1.49 ± 0.04 bits	n/a	n/a

4. Determining intrinsic mutual information

4.1. **Intrinsic and extrinsic contributions to gene expression noise.** It is well-known that gene expression is noisy in the sense that a group of genetically identical cells exposed to the same perturbation or signal will exhibit significant cell-to-cell variability in their gene expression responses. In terms of information transduction this leads to a loss of information.

The sources of gene expression noise can be classified into two broad sources: intrinsic and extrinsic noise (Elowitz *et al,* 2002; Swain *et al,* 2002; Hilfinger & Paulsson, 2011). To experimentally distinguish these two sources of gene expression variability, the dual-reporter system was developed (Elowitz *et al,* 2002). Using diploid yeast, equivalent CFP and YFP gene expression reporters are placed on the homologous chromosomes at the same locus. Thus, the two reporters are in identical genetical contexts. Extrinsic noise is then defined as variability caused by the shared intracellular environment, such as differences in the number of RNA Pol II complexes, Msn2 molecules, ribosomes or cell cycle phase between cells, which affect both CFP and YFP within the same cell equally. The remaining variability, which is not accounted for by the shared environment, is then defined as intrinsic noise and could stem from stochastic TF binding or chromatin remodeling at individual promoters (Elowitz *et al,* 2002; Swain *et al,* 2002).

Mathematically, noise is defined as $\eta^2 = \frac{\sigma^2}{\mu^2}$. Using dual CFP and YFP reporters, gene expression noise can be decomposed into its intrinsic and extrinsic parts:

$$\eta^2_{\text{total}} = \frac{\langle \text{CFP}^2 + \text{YFP}^2 \rangle - 2\langle \text{CFP} \rangle \langle \text{YFP} \rangle}{2\langle \text{CFP} \rangle \langle \text{YFP} \rangle} \tag{4.1}$$

$$\eta^2_{\text{extrinsic}} = \frac{\langle \text{CFP} \cdot \text{YFP} \rangle - \langle \text{CFP} \rangle \langle \text{YFP} \rangle}{\langle \text{CFP} \rangle \langle \text{YFP} \rangle} \tag{4.2}$$

$$\eta^2_{\text{intrinsic}} = \frac{\langle (\text{CFP} - \text{YFP})^2 \rangle}{2\langle \text{CFP} \rangle \langle \text{YFP} \rangle} \tag{4.3}$$

Finally, we note that $\eta^2_{\text{tot}} = \eta^2_{\text{int}} + \eta^2_{\text{ext}}$.

4.2. **Effect of extrinsic noise on mutual information.** Ideally, mutual information and the information transduction capacity of a promoter would be measured in the same cell. That is, using I_{AM} as an example, the ideal experiment would involve exposing the same cell to different amplitudes of Msn2 translocation and measuring a gene expression response each time and repeating this thousands of times in each single cell. However, this is not experimentally feasible: each experiment takes ~ 3 hours and there is the issue of adaptation, which means the first response will be different from the second.

Instead, to measure I, we must take populations of cells and expose them to a single amplitude of Msn2 translocation and repeat this experiment multiple times to obtain enough cells for each amplitude and to obtain data for different conditions (i.e. different amplitudes). However, this means that the measured I might be an underestimate.

To illustrate this point, consider the cell cycle. It is known that the cell cycle can affect gene expression variability (Zopf *et al,* 2013). Thus, when we measure the gene expression response to a Msn2 signal across a cell population with unsynchonized cell cycle phases, part of the variability that we observe is really due to cell cycle variability, rather than anything due to the PKA/Msn2 signaling pathway. While it is possible to determine the cell cycle phase and therefore condition each cell's response on the initial cell cycle phase, this is very labor intensive, but more importantly the cell cycle is only one of a huge number of known and unknown extrinsic factors. Thus, we need a method that can correct for all extrinsic effects, without having to specify each individual source of extrinsic noise, and estimate a purely intrinsic I.

The importance of extrinsic effects on single cell dose-responses was highlighted in recent work from Toettcher, Lim and Weiner (Toettcher *et al,* 2013). Using optogenetics to control Ras activation (input) and microscopy to measure nuclear translocation of ERK (output), Toettcher *et al.* was able to measure full dose-responses in single cells because of the rapid response time and the lack of adaptation of their system. The key finding was that the single cell dose-responses exhibited much lower variability than the dose-response obtained from averaging over a population of cells. Rephrasing this in an information theoretic framework, this work experimentally demonstrates that single cells condition on extrinsic factors and that I measured across a cell population could be a serious underestimate of the true I.

4.3. **An algorithm to estimate the intrinsic mutual information.** Using the dual CFP/YFP reporters in the same cell, we can calculate intrinsic and extrinsic noise. To determine the intrinsic maximal mutual information, I_{int}, we need to determine the full gene expression probability distribution in the absence of extrinsic noise. This is an intractable problem and the intrinsic noise component has to be carefully interpreted (Hilfinger & Paulsson, 2011; Shahrezaei *et al,* 2008). Here we present an algorithm to infer an approximate I_{int}. The approach is outlined in Figure 6. We stress that this approach is approximate and that it is not possible to analytically determine the gene expression distribution in the absence of extrinsic noise. Nonetheless, comparisons using stochastic simulations validate the approach and indicate that the error is small.

In the following we go through each step in the algorithm. First, for each condition we fit the raw empirical distribution of YFP gene expression responses to a gamma distribution. That is, if X is the random variable representing the YFP response, then $X \sim \Gamma(a, b)$. We choose a gamma distribution because it is a highly flexible distribution with positive support. More importantly, a gamma distribution has been justified in terms of theoretical stochastic models of gene expression (Friedman *et al,* 2006), statistical inference approaches (Zechner *et al,* 2014) and shown to be able to fit 1009 out of 1018 protein distributions across the *E. coli* proteome (Taniguchi *et al,* 2010). Finally, we found that our empirical YFP responses were well-fit by gamma distributions. An example of the gamma fit to a raw empirical YFP distribution is shown in Figure 6 (black fit to red

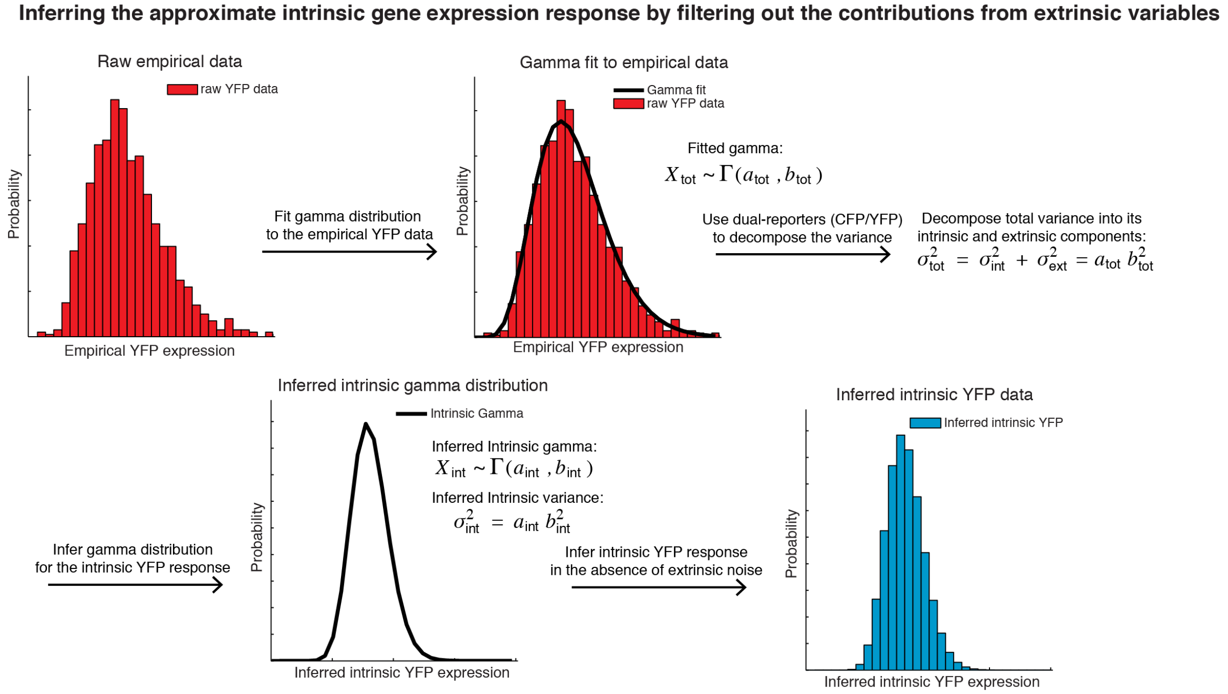

FIGURE 6. **Inferring an estimate of I_{int}**
The raw empirical YFP distribution (red) is fitted to a gamma distribution. While keeping the mean constant, a new gamma distribution is inferred where the variance is now equal to the intrinsic variance. This distribution is then used for calculating the I_{int}.

histogram). An obvious case where a single gamma distribution would be insufficient would be a bimodal distribution, but we do not observe any bimodal responses.

Let $X_{\text{tot}} \sim \Gamma(a_{\text{tot}}, b_{\text{tot}})$ represent the YFP response in the presence of extrinsic noise and $X_{\text{int}} \sim \Gamma(a_{\text{int}}, b_{\text{int}})$ the YFP response in the absence of extrinsic noise. To determine a_{int} and b_{int}, we first calculate the intrinsic variance from the empirical CFP and YFP data according to equation (4.3):

$$\sigma^2_{\text{int}} = \eta^2_{\text{int}}\langle\text{CFP}\rangle\langle\text{YFP}\rangle = \frac{1}{2}\langle(\text{CFP} - \text{YFP})^2\rangle \tag{4.4}$$

In addition to assuming that both X_{tot} and X_{int} are gamma distributed, we also assume that the mean is unchanged. For gamma distributions, $E(X) = ab$ and $\text{Var}(X) = ab^2$. Thus, we require $a_{\text{tot}}b_{\text{tot}} = a_{\text{int}}b_{\text{int}}$. If we define $k = \frac{\sigma^2_{\text{tot}}}{\sigma^2_{\text{int}}}$, we therefore get:

$$a_{\text{int}} = k a_{\text{tot}} \tag{4.5}$$

$$b_{\text{int}} = \frac{b_{\text{tot}}}{k} \tag{4.6}$$

Thus, if $X \sim \Gamma(a, b)$ then $X_{\text{int}} \sim \Gamma\left(a\frac{\sigma^2_{\text{tot}}}{\sigma^2_{\text{int}}}, b\frac{\sigma^2_{\text{int}}}{\sigma^2_{\text{tot}}}\right)$, where σ^2_{int} is defined in equation (4.4). We use the MATLAB function `gamfit` to fit a gamma distribution to the raw data and calculate a and b.

Next and using *HXK1* with respect to amplitude modulation as an example, we then repeat the above procedure for each amplitude and discretize the intrinsic gamma distribution using the same bins as for the empirical YFP distribution. An example is shown in Figure 7.

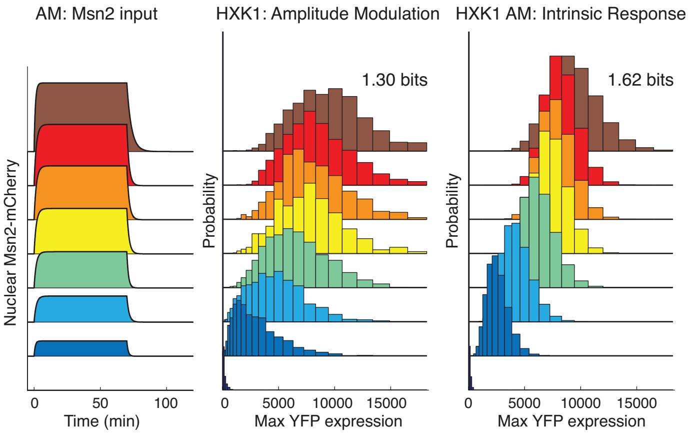

Figure 7. **Inferring the approximate intrinsic gamma distribution**
Using *HXK1* with respect to amplitude modulation as an example, the empirical YFP response distributions are plotted next to the intrinsic gamma distribution. The empirical I_{raw} and the inferred approximate I_{int} are listed as well.

Finally, having determined the intrinsic gamma distribution, I_{int} is calculated according to equation (2.4) using the Blahut-Arimoto algorithm as previously. As shown in Figure 7 the intrinsic response distributions for *HXK1* are significantly more narrow and I_{int} significantly greater than I_{raw}.

It is important to note that it is not possible to analytically determine the intrinsic distributions from filtering extrinsic noise out of the empirical distribution (Hilfinger & Paulsson, 2011). Here we approximate both the total and intrinsic distributions as gamma distributions and make several important assumptions that cannot rigorously be justified. In the next section we test the algorithm on simulated data to determine how well the algorithm can estimate the true I_{int}.

4.4. **Testing the algorithm with simulated data and linear gene expression models.** To systematically test the algorithm and estimate the error in the estimation of I_{int} we took the following approach. For the 5 linear models shown in Figure 8, we used the Gillespie algorithm (Gillespie, 1977) to simulate stochastic gene expression, with two reporter genes per cell, for thousands of cells and several conditions (e.g. different TF amplitudes), with or without extrinsic noise. We model extrinsic noise by letting rate constants differ between cells (Shahrezaei *et al,* 2008; Zechner *et al,* 2014). We then apply the algorithm to the data with extrinsic noise, calculate the inferred I_{int} and compare this to I_{int} obtained from the data with only intrinsic noise. Below, we go through each step in detail.

To test the generality of our approach, we test it using five different gene expression models (Figure 8). Model 1 and 2 are versions of the classic 2-state random telegraph model (Ko 1991; Peccoud & Ycart, 1995) with transcription either dependent or independent of [TF(t)], respectively.

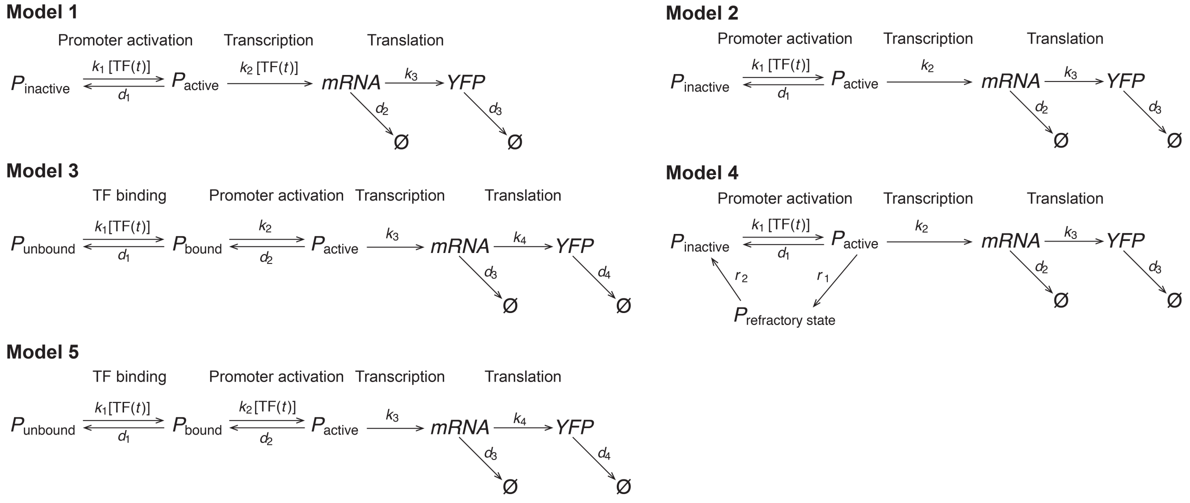

FIGURE 8. **Linear gene expression models**
The I_{int} algorithm was tested using data simulated with the 5 linear models above.

Model 3 (Zechner *et al,* 2014) and model 5 (Rieckh & Tkacik, 2014) are 3-promoter state versions of the random telegraph model and model 4 includes a refractory state (Zechner *et al,* 2014).

In the following we will use model 1 to illustrate the steps. To model multiple conditions, we vary the transcription rate from 0.025 min^{-1} to 3 min^{-1} in 10 steps. Previous approaches have used the assumption that all extrinsic noise comes from translation (Zechner *et al,* 2014). Since for our purpose another direct source of extrinsic variability is variation in Msn2 levels between cells, we model extrinsic noise as coming from translation and $[\text{TF}(t)]$. Thus, for each simulated cell we pick k_3 (model 1) and $[\text{TF}(t)]$ from a gamma distribution. Since the upper bound for a gamma distribution is ∞, it will occasionally happen that unphysically large or small values of k_3 (model 1) and $[\text{TF}(t)]$ are picked and we therefore set a lower and upper threshold of half and twice the mean, respectively. If values above or below this threshold are picked, we re-pick until a physically plausible value is picked. Furthermore, to both simulate cases where intrinsic and extrinsic noise dominates we repeat the simulations where k_3 and $[\text{TF}(t)]$ are picked from a gamma distribution with low variance (such that intrinsic noise dominates) and from a gamma distribution with high variance (such that extrinsic noise dominates). Finally, to find I in the absence of extrinsic noise, we also perform simulations where $[\text{TF}(t)]$ and all the rate parameters have fixed values. This gives the true I_{int}. For each condition we use 2000 iterations, corresponding to 2000 simulated cells, over 10 conditions, corresponding to 10 amplitudes of a 70 min pulse. To test generality, we simulate both a slower ($k_1 = d_1 = 0.1$ min^{-1} for model 1) and a faster ($k_1 = d_1 = 0.5$ min^{-1} for model 1) promoter (slower promoters exhibit substantially higher gene expression noise and therefore, all other things being equal, have lower I (Hansen & O'Shea, 2013)).

Figure 9 shows an example of a typical model simulation over the 10 conditions. The true I_{int} (center), is obtained from simulations without extrinsic noise (1.426 bits). The algorithm is then applied to data with extrinsic noise (left) and used to infer an estimate (1.415 bits) of the true I_{int} (right).

Figure 10 summarizes the results from simulations over 5 different conditions for each of the 5 models. Figure 10A shows I with and without extrinsic noise. The parameters were chosen such that I would be in the 1-2 bit range as we observe experimentally. Figure 10B shows the error in the inferred I_{int} for each of the 4 conditions with extrinsic noise. The algorithm generally

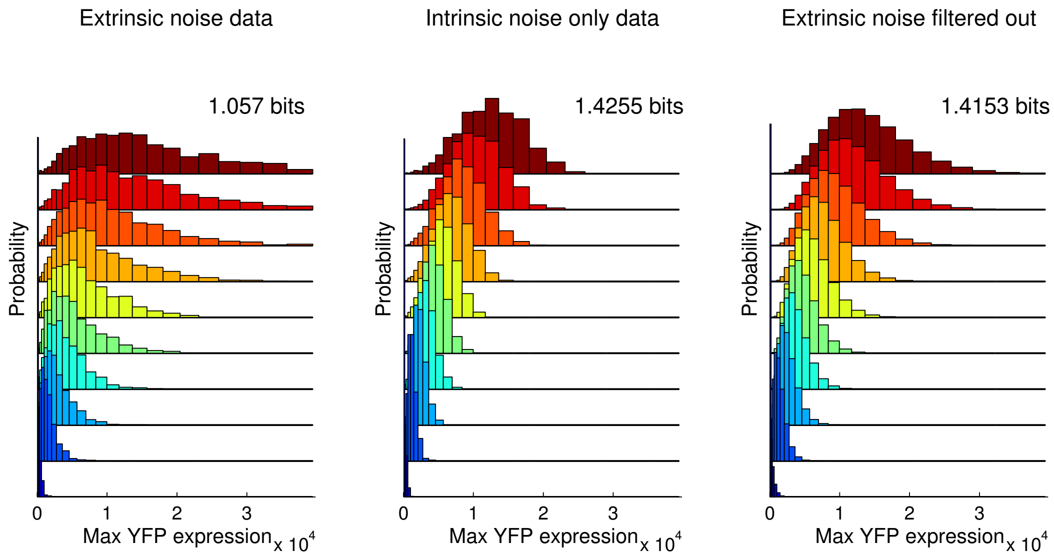

FIGURE 9. **Example of the algorithm on simulated data using a linear model**

Using model 1 and a slow promoter, data with a high level of extrinsic noise was simulated over 10 conditions (left), such that extrinsic noise dominates over intrinsic noise at higher expression. The same system was simulated without extrinsic noise (center) and the corresponding I calculated. Finally, the algorithm was applied to the data with extrinsic noise (left) and intrinsic distributions were inferred (right) and used to estimate I_{int}. As can be seen, the inferred I_{int} is a slight underestimate.

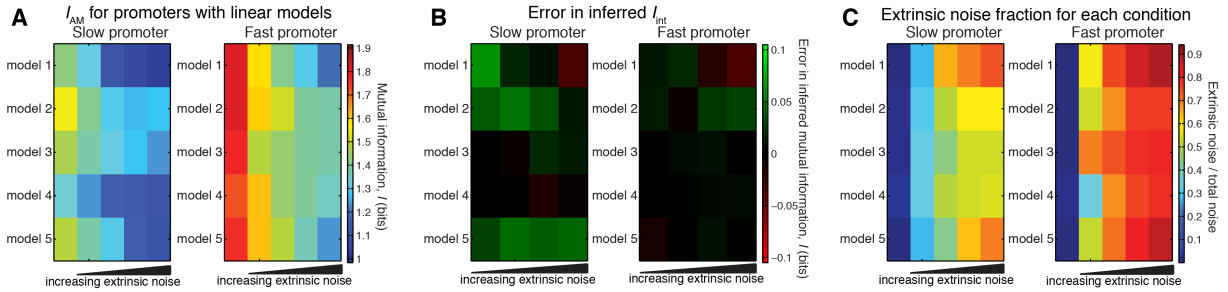

FIGURE 10. **Testing the algorithm using simulated data and linear models**

A) For each of the 5 linear models, two promoters (slow and fast) were simulated without and with increasing amounts of extrinsic noise and I determined. **B)** For the 4 conditions with extrinsic noise, the error in the estimated I_{int} was determined for each promoter. **C)** The fraction of the total noise that is extrinsic for each simulated condition (shown is the fraction for the amplitude that yields maximal expression)

underestimates the true I_{int}. However, the error is very small. In all cases, the error is less than 5% and the average error is less than 2%. This should be compared with an error from the simulations of around 1%. Finally, as shown in Figure 10C we choose the level of extrinsic noise (shown only for the maximum expression condition) to vary over a range of levels. For the experimental data, extrinsic noise always dominates at the 70 min 3 μM 1-NM-PP1 condition, but to different extents:

it vastly dominates for p*SIP18* mut B, but less so for *SIP18*. Thus, the extrinsic noise was similarly varied for the simulations.

4.5. **Testing the algorithm with simulated data and nonlinear gene expression models.** To exhaustively test the algorithm, we repeated the above approach of simulating 5 models with and without extrinsic noise and then applying the algorithm to the extrinsic noise data and testing how well it matches the true I_{int} using also nonlinear models. To fully account for the nonlinear dependence of gene expression on the Msn2 amplitude, nonlinear functions such as the Hill function are necessary (Hansen & O'Shea, 2013). Thus, we consider the same 5 models as in Figure 8 but this time with a nonlinear Hill function-type dependence on the nuclear Msn2 concentration as shown in Figure 11.

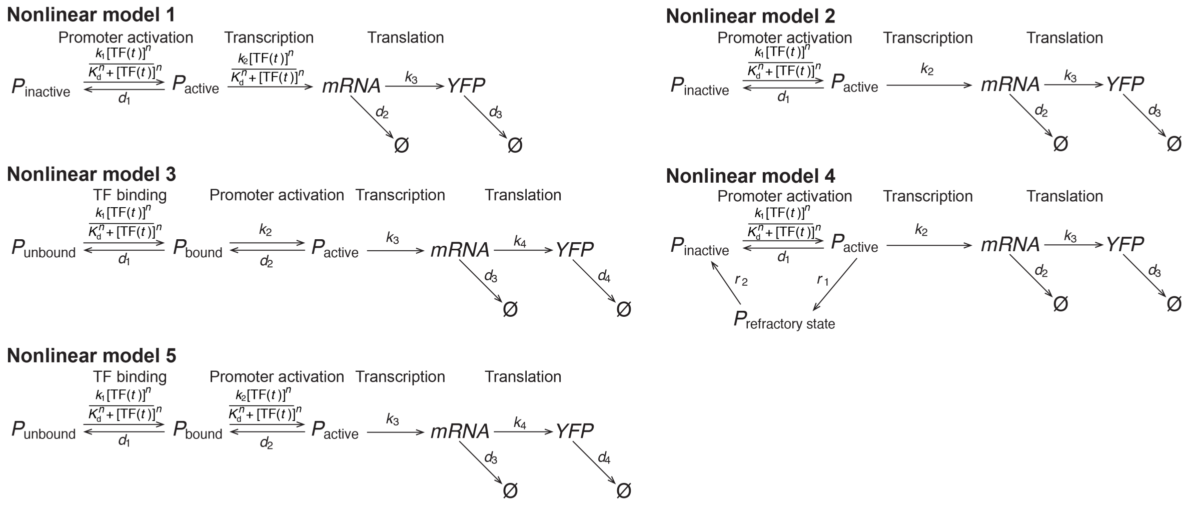

FIGURE 11. **Nonlinear gene expression models**
The I_{int} algorithm was further tested using data simulated with the 5 nonlinear models above.

Without repeating the previous section, for the nonlinear models we followed exactly the same approach as for the linear model simulations. For the Hill function we picked, $K_d = 1.5$ and $n = 1.5$ (this level of moderate cooperativity is consistent with our previous observations (Hansen & O'Shea, 2013)) and we now allow the TF concentration to increase with each of the 10 conditions up until a final value of 10 in the same arbitrary units as the K_d is given in. As before, we model extrinsic noise by picking the TF concentration and the translation rate (k_3 in model 1) from a gamma distribution and for each model we consider both a slow and a fast promoter. As before, we summarize the simulation results as a heatmap (Figure 12).

As shown in Figure 12, the error is always small also for the nonlinear models. In fact, as for the linear models the algorithm tended to slightly underestimate the true I_{int}, but again, the error was always less than 5% and the average error was less than 2%.

4.6. **Comments on the algorithm.** Taken together, we have considered 5 linear and 5 nonlinear gene expression models and a slow and a fast promoter for each and in each case four different levels of extrinsic noise. In every one of these 80 simulations we find that the error is always below 5% (in bits) and the mean error is less than 2%. As mentioned under most conditions the extrinsic noise can be precisely measured, but it is not possible to obtain the full intrinsic probability distribution (Hilfinger & Paulsson, 2011). Yet, based on the model simulations our algorithm seems to work very well and gives negligible error. One possibility is that the various approximations cancel each other

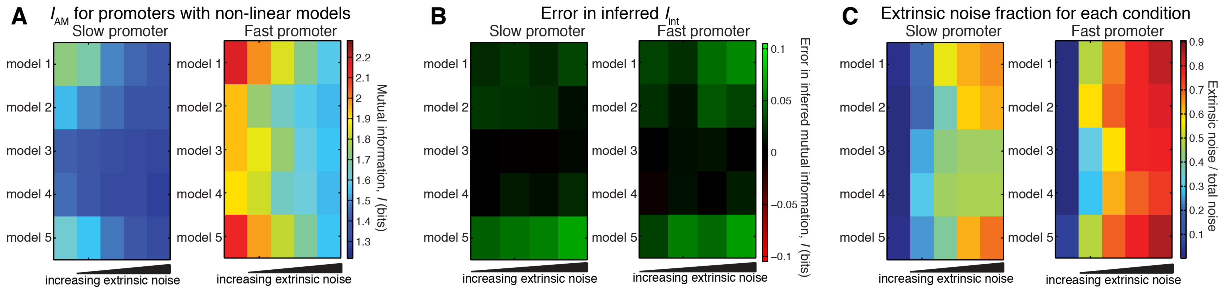

FIGURE 12. **Testing the algorithm using simulated data and nonlinear models**

A) For each of the 5 nonlinear models, two promoters (slow and fast) were simulated without and with increasing amounts of extrinsic noise and I determined. **B)** For the 4 conditions with extrinsic noise, the error in the estimated I_{int} was determined for each promoter. **C)** The fraction of the total noise that is extrinsic for each simulated condition (shown is the fraction for the amplitude that yields maximal expression)

out. Regardless, we do not make claims about the precise I_{int} of a specific Msn2-dependent promoter, but rather roughly of what order it is. E.g. for *SIP18*, I_{int} was inferred to be ca. 1.51 ± 0.07 bits. Given the error range of our algorithm, we cannot with certainty say what the exact value it, but we can say that it is almost certainly less than 2 bits and therefore that signal intensity information cannot be amplitude transmitted through the *SIP18* gene without significant associated error.

4.7. Computing I_{int} with empirical data.

To estimate I_{int} using empirical data we apply the algorithm as explained above and shown in Figure 7. The results are shown in the table below:

Promoter	I_{raw}	I_{int}
HXK1: AM	1.30 ± 0.02 bits	1.61 ± 0.02 bits
HXK1: FM	1.11 ± 0.01 bits	1.36 ± 0.02 bits
SIP18: AM	1.21 ± 0.03 bits	1.51 ± 0.07 bits
SIP18: FM	0.52 ± 0.06 bits	0.76 ± 0.07 bits
pSIP18 mut A: AM	1.42 ± 0.01 bits	1.59 ± 0.02 bits
pSIP18 mut A: FM	0.88 ± 0.02 bits	0.96 ± 0.02 bits
pSIP18 mut B: AM	1.55 ± 0.01 bits	2.00 ± 0.02 bits
pSIP18 mut B: FM	1.39 ± 0.01 bits	1.69 ± 0.02 bits

To estimate the error in I_{int}, we follow the same approach as for the empirical I_{raw} shown in Figure 1 to correct for bias due to binning and undersampling. That is, we perform jackknife sampling to estimate C_{unbiased} (equation (2.5)) using 15-35 bins, inclusive, and report I_{int} as the mean and the error as the standard deviation.

5. REFERENCES

Arimoto, S. (1972). An algorithm for computing the capacity of arbitrary discrete memoryless channels. *IEEE Trans. Inform. Theory.*

Blahut, R. E. (1972). Computation of channel capacity and rate-distortion functions. *IEEE Trans. Inform. Theory.*

Bowsher, C.G., Swain, P.S. (2014). Environmental sensing, information transfer, and cellular decision making. *Curr Opin Biotech*, 28, 149-155.

Cheong, R., Rhee, A., Wang, C. J., Nemenman, I., Levchenko, A. (2011). Information transduction capacity of noisy biochemical signaling networks. *Science*, 334, 354-358.

Cover, T. M., Thomas, J. A. (2006). Elements of information theory. *Wiley.*

Elowitz, M. B., Levine, A. J., Siggia, E. D., Swain, P. S. (2002). Stochastic gene expression in a single cell. *Science*, 297, 1183-1186.

Friedman, N., Cai, L., Xie, X. S. (2006). Linking stochastic dynamics to population distribution: an analytical framework of gene expression. *Physical review letters*, 97, 168302.

Gillespie, D. T. (1977). Exact stochastic simulation of coupled chemical reactions. *The journal of physical chemistry*, 81, 2340-2361.

Hansen, A. S., O'Shea, E. K. (2013). Promoter decoding of transcription factor dynamics involves a trade-off between noise and control of gene expression. *Molecular systems biology*, 9.

Hilfinger, A., Paulsson, J. (2011). Separating intrinsic from extrinsic fluctuations in dynamic biological systems. *Proceedings of the National Academy of Sciences*, 108, 12167-12172.

Ko M. S. H. (1991). A stochastic model for gene induction. *Journal of Theoretical Biology*, 153, 181-194.

Levchenko, A., Nemenman, I. (2014). Cellular noise and information transmission. *Curr Opin Biotech*, 28, 156-164.

Peccoud J., Ycart B. (1995). Markovian Modeling of Gene Product Synthesis. *Theor Popul Biol*, 48, 222-234.

Rieckh, G., Tkacik, G. (2014). Noise and information transmission in promoters with multiple internal states. *Biophysical journal*, 106, 1194-1204.

Shahrezaei, V., Ollivier, J. F. Swain, P. S. (2008), Colored extrinsic fluctuations and stochastic gene expression. *Molecular Systems Biology*, 4.

Shannon, C. E. (1948), A mathematical theory of communication. *Bell System Technical Journal*, 27, 379-423 and 623-656.

Slonim, N., Atwal, G. S., Tkacik, G., Bialek, W. (2005). Information-based clustering. *Proceedings of the National Academy of Sciences of the United States of America*, 102, 18297-18302.

Strong, S. P., Koberle, R., de Ruyter van Steveninck, R. R., Bialek, W. (1998). Entropy and Information in Neural Spike Trains. *Physical Review Letters*, 80, 197-200.

Swain, P. S., Elowitz, M. B., Siggia, E. D. (2002). Intrinsic and extrinsic contributions to stochasticity in gene expression. *Proceedings of the National Academy of Sciences*, 99, 12795-12800.

Taniguchi, Y., Choi, P. J., Li, G. W., Chen, H., Babu, M., Hearn, J., Emili, A., Xie, X. S. (2010). Quantifying E. coli proteome and transcriptome with single-molecule sensitivity in single cells. *Science*, 329, 533-538.

Toettcher, J. E., Weiner, O. D., Lim, W. A. (2013). Using optogenetics to interrogate the dynamic control of signal transmission by the Ras/Erk module. *Cell*, 155, 1422-1434.

Zechner, C., Unger, M., Pelet, S., Peter, M., Koeppl, H. (2014). Scalable inference of heterogeneous reaction kinetics from pooled single-cell recordings. *Nature methods*, 11, 197-202.

Zopf, C. J., Quinn, K., Zeidman, J., Maheshri, N. (2013). Cell-cycle dependence of transcription dominates noise in gene expression. *PLoS computational biology*, 9, e1003161.
